# From Patient-Specific Mathematical Neuro-Oncology to Precision Medicine

**DOI:** 10.3389/fonc.2013.00062

**Published:** 2013-04-02

**Authors:** A. L. Baldock, R. C. Rockne, A. D. Boone, M. L. Neal, A. Hawkins-Daarud, D. M. Corwin, C. A. Bridge, L. A. Guyman, A. D. Trister, M. M. Mrugala, J. K. Rockhill, K. R. Swanson

**Affiliations:** ^1^Department of Neurological Surgery, Northwestern UniversityChicago, IL, USA; ^2^Brain Tumor Institute, Northwestern UniversityChicago, IL, USA; ^3^Department of Pathology, University of WashingtonSeattle, WA, USA; ^4^Department of Medical Education and Biomedical Informatics, University of WashingtonSeattle, WA, USA; ^5^Department of Radiation Oncology, University of WashingtonSeattle, WA, USA; ^6^Department of Neurology, University of WashingtonSeattle, WA, USA; ^7^Department of Applied Mathematics, University of WashingtonSeattle, WA, USA

**Keywords:** glioma, mathematical modeling, patient-specific, clinical modeling, personalized medicine, individualized health care

## Abstract

Gliomas are notoriously aggressive, malignant brain tumors that have variable response to treatment. These patients often have poor prognosis, informed primarily by histopathology. Mathematical neuro-oncology (MNO) is a young and burgeoning field that leverages mathematical models to predict and quantify response to therapies. These mathematical models can form the basis of modern “precision medicine” approaches to tailor therapy in a patient-specific manner. Patient-specific models (PSMs) can be used to overcome imaging limitations, improve prognostic predictions, stratify patients, and assess treatment response *in silico*. The information gleaned from such models can aid in the construction and efficacy of clinical trials and treatment protocols, accelerating the pace of clinical research in the war on cancer. This review focuses on the growing translation of PSM to clinical neuro-oncology. It will also provide a forward-looking view on a new era of patient-specific MNO.

## The Clinical Challenge of Patient-Specific Prognosis and Treatment Response

Gliomas are heterogeneous primary brain tumors that exhibit widely varying phenotypes even within the same histological grade (Louis et al., 2007). These tumors are characterized by proliferating and invading adjacent normal brain tissue, resulting in a significant clinical challenge and generally high morbidity and mortality. Despite advances in medical imaging technologies, surgery, radiation therapy, and chemotherapies over the last several decades, the standard of care for newly diagnosed malignant gliomas does not reflect individual differences (Stupp et al., 2007; Nishikawa, 2010). Prognosis for glioma patients has hardly changed in over 50 years of cancer research. The incorporation of patient-specific measures of prognosis and treatment response allows tailor therapies for each patient.

### Invisible, inherent, invasion

**Figure 1:**
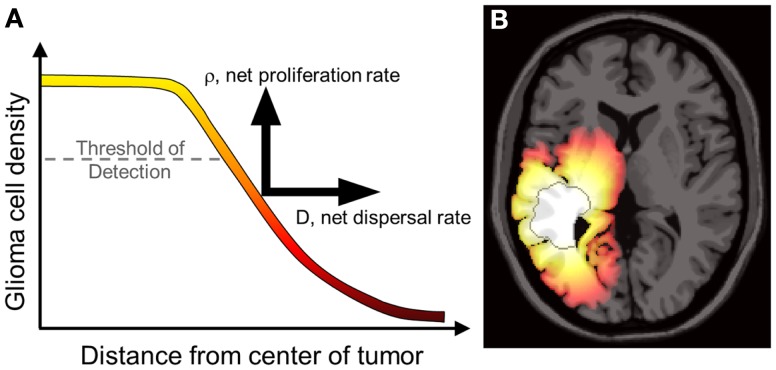
**(A)** Concentrations of tumor cells contributing to a gradient of diffusely invading glioma cells extending well beyond the threshold of detection. The PI model characterizes the net rates of growth and invasion of the glioma cells contributing to this overall profile, a sum of individual cell behaviors. Swanson et al. have demonstrated that *D* and ρ can be calculated on a patient-specific basis and can vary widely, even for patients within the same histological grade (Harpold et al., 2007; Swanson et al., 2008a; Szeto et al., 2009b; Wang et al., 2009; Rockne et al., 2010). **(B)** A simulation of the reaction-diffusion mathematical model on an anatomically accurate brain phantom (Cocosco et al., 2004) with differential motility in gray and white matter as proposed by Swanson (1999). The MRI-detectable edge of the lesion is superimposed as a dark gray contour emphasizing the extent of invasion well beyond the threshold of detection. From Wang et al. (2009) with permission from Cancer Research.

The most significant characteristic of gliomas of all grades is their diffuse invasion into the normal-appearing brain as seen in gross pathology and histological specimens. To reduce the morbidity of extensive biopsies, primary clinical assessment and staging of gliomas relies on non-invasive radiographic imaging such as magnetic resonance imaging (MRI) and computed tomography (CT). However, neither of these imaging techniques quantifies the full extent of tumor invasion due to the inherent limits of detection, as illustrated in Figure 1. Furthermore, post-treatment surveillance for recurrence and progression is based on these same imaging technologies (Swanson, 1999; Harpold et al., 2007; Szeto et al., 2009b; Pallud et al., 2010). The inability to completely quantify the glioma cell population (invisibility) and diffuse extension into normal-appearing brain (invasion) along with wide heterogeneity between and within patients makes a personalized approach to treatment and measuring response difficult but necessary.

## Why Use Mathematical Modeling?

Currently, prognosis in glioma patients is based upon retrospective analyses of groups of patients with similar histopathological characteristics (Louis et al., 2007). This approach is unsatisfying because of the heterogeneity of disease phenotypes within each larger histological category as well as the lack of insight into treatment modalities that may most benefit an individual patient. Mathematical models are used to bridge this gap and have been used to illustrate individual differences in the dynamics of glioma growth and response to therapy in a research setting, with the potential for clinical translation on the horizon. To optimize individual treatment protocols, physicians and scientists require tools to evaluate the relative benefit obtained for each patient. The subset of patients with the most aggressive tumors (and inherently worst prognosis) stand to benefit the most from the shift from one-size-fits-all treatment to a patient-specific approach, and models allow for the prospective identification of these patients.

Mathematical models already have a significant impact on clinical practice, as they are widely integrated into medical imaging technologies (e.g., Carson et al., 1998). Furthermore, mathematical models are found throughout the biological sciences, with one of the most common applications being population models for a single species (e.g., Murray, 2002). Yet it can be quite a leap for both the basic scientist and clinician to embrace the idea that a relatively simple mathematical model might shed light on such a complex disease process as malignant glioma. Some believe that gliomas are so biologically complex and heterogeneous that no model could provide insight into the inherent nature of disease. On the contrary, as clinical oncology strives to provide personalized management of cancer, mathematical models are playing a pivotal role in providing insight into disease growth, treatment response, and ultimately building the framework for precision medicine (Council, 2011).

### Patient-specific mathematical neuro-oncology

The term Mathematical Oncology was coined in 2003 to reflect the burgeoning synergy between mathematical modeling techniques, cancer research, and clinical oncology (Gatenby and Maini, 2003). The term has been subsequently refined to Integrated Mathematical Oncology to emphasize the feedback that emerges through the integration of mathematics and oncology (Anderson and Quaranta, 2008). This review focuses on the patient-specific applications of Mathematical *Neuro*-Oncology (MNO) to provide predictive insight onto glioma prognosis and treatment response in individual patients. Although the field of neuro-oncology is broad and encompasses many distinct neoplasms, to date, much of the literature has been devoted to the presentation of models and methodologies for estimating glioma growth from medical imaging and other clinical data.

In this paper, the focus is on models that will truly enable “precision medicine.” Thus the discussion below will revolve around only models the authors believe are patient-specific in nature and have in some way been subjected to validation tests. Specifically we have reviewed reaction-diffusion models such as those championed by Swanson, which take a macroscopic perspective of gliomas as a continuum of tumor cell concentration.

## Individual Tumor Growth Kinetics: A Predictable Pattern

Gliomas of all histologic grades exhibit a constant velocity of the mean tumor radius if left untreated, resulting in predictable pattern of linear radial growth (Swanson and Alvord, 2002; Mandonnet et al., 2003, 2008; Pallud et al., 2006). Mandonnet et al. (2003) demonstrated this in 27 untreated low-grade gliomas (LGG) followed with serial routine MRI for up to 15 years. Despite the anatomic heterogeneity in tumor growth, the average radius of each glioma increased linearly with time, with rates ranging from 1 to 4 mm/year. Furthermore, the velocity of linear radial expansion was shown to predict time to malignant progression (Hlaihel et al., 2010) and is a significant predictor of survival (Pallud et al., 2006; Swanson et al., 2008a).

Constant linear radial growth is also seen in a rare example of an *untreated* high grade glioma known as glioblastoma multiforme (GBM). In this case a 75-year-old female presented to the emergency department with a complex partial seizure (CPS), prompting imaging that revealed a large tumor. She refused medical advice to undergo a biopsy to establish a diagnosis and subsequent treatment but allowed multiple imaging observations (Swanson and Alvord, 2002). The patient was found to have a GBM on autopsy, and the serial imaging revealed a consistent linear radial growth pattern.

### The proliferation-invasion model of glioma growth

**Figure 2:**
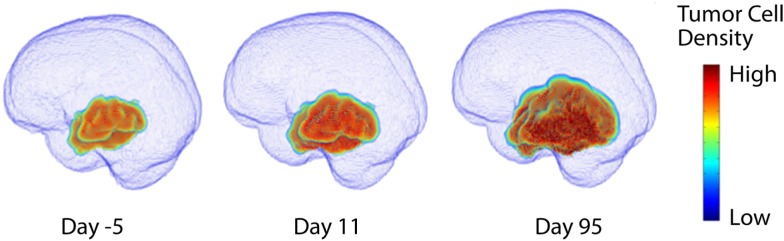
**Three dimensional simulation of diffuse tumor invasion and proliferation predicted by the PI model which accounts for differential motility of tumor cells in gray and white matter**. Malignant glioma cells can migrate up to 100-fold faster in white matter than in gray, characterizing the extent of invisible subclinical disease.

In the early 1990s, the research groups of Murray and Alvord developed a mathematical model to describe the diffuse infiltration and proliferation of glioma cells in the complex anatomy of the human brain (Figure 2). This model can be described in words as the rate of change of tumor cell density in time is equal to the net migration of tumor cells plus the net proliferation of tumor cells. Mathematically, the model is a partial differential equation with two parameters: net rates of migration (*D*, mm^2^/year) and proliferation (ρ, year^−1^), both of which can be calculated on a patient-specific basis using routine clinical imaging prior to treatment.

$$\begin{array}{llll} \overset{\begin{array}{c} \text{rate of change of tumor}\\ \text{cell density over time} \end{array}}{\overset{︷}{\frac{\partial c}{\partial t}}}=\overset{\begin{array}{c} \text{net migration of tumor}\\ \text{cells} \end{array}}{\overset{︷}{\nabla \cdot \left(D\left(x\right)\nabla c\right)}}+\overset{\begin{array}{c} \text{net proliferation}\\ \text{of tumor cells} \end{array}}{\overset{︷}{\rho c\left(1-\frac{c}{K}\right)}} & & & \end{array}$$

This “proliferation-invasion” model (PI model) of glioma growth and infiltration is similar to Fisher’s equation which yields the same predictable pattern of linear radial growth observed in low and high grade gliomas (Fisher, 1937). The velocity of growth predicted by this equation relates the velocity of radial growth to the square root of the product of net dispersal (*D*) and proliferation (ρ) parameters, $v=\sqrt{4D\text{ρ}}$. This relationship combined with an “invisibility index” (*D*/ρ) relating rates of invasion and proliferation provides two equations and two unknown parameters, tuning the PI model to patient-specific growth. The PI model can further incorporate differential motility of glioma cells through gray and white matter of the brain, providing predictions of diffuse tumor invasion through the regions of the brain that are specific to the patient’s tumor (Figure 2). This simple model has served as a foundation for patient-specific MNO and provided numerous insights into clinical behaviors such as survival outcome (Pallud et al., 2006; Swanson et al., 2008b; Wang et al., 2009; Rockne et al., 2010), hypoxia development (Szeto et al., 2009b), response to surgical resection (Swanson et al., 2008b), chemo- and radiation therapies (Rockne et al., 2010), biological aggressiveness (Szeto et al., 2009a; Ellingson et al., 2010b), and to date is the single most applied patient-specific clinical scale model for glioma growth and response to therapy. Extensions to this model include consideration of anisotropic growth in white matter tracts (Jbabdi et al., 2005). Mass effect and mechanical constraints of anatomical structures such as the skull, have been included to refine spatial agreement with patient scans (Clatz et al., 2005). Giatili and Stamatakos (2012) add adiabatic Neumann boundary conditions to more realistically model the boundary imposed by the skull. These efforts show room for further model development, but have yet to be applied to a patient population as large as that modeled by the PI model.

Advanced imaging techniques such as diffusion weighted MRI (DWI), MRI Spectroscopy, and Diffusion Tensor (DT) MRI have been used to suggest techniques for estimating patient-specific parameters *D* and ρ (Ellingson et al., 2010a; Konukoglu et al., 2010). Based on the assumption that the apparent diffusion coefficient (ADC), which measures magnitude of diffusion of water, is negatively proportional to tumor cell density, Ellingson et al. proposes that *D* and ρ of the PI model, modified such that proliferation is exponential and not saturated in a saturated environment can be estimated on a patient-specific basis using three ADC imaging time points. Ellingson et al. applied this methodology and found a stratification of *D* and ρ with histologic grading which compares well with the previous estimates for high grade gliomas but differs significantly from the estimates for low-grade proliferation and invasion kinetics (Harpold et al., 2007). This difference may be explainable by the fact that the correlations between ADC and overall tissue cell density utilized by the Ellingson approach incorporates both normal and malignant cell densities while the PI model is only tracking the glioma cell density. Further exploiting opportunities provided by diffusion MRI, Konukoglu et al. (2010) uses DT-MRI to inform model predictions of separate diffusion rates in gray and white matter.

### “Go or grow” hypothesis

Experimental data suggests that tumor states of proliferative and invasive capacity are mutually exclusive (Giese et al., 2003). The “go or grow” hypothesis has influenced mathematical models analyzing how the rates of switching between proliferative and migratory phenotypes affect macroscopic tumor growth (Gerlee and Nelander, 2012). Hatzikirou et al. (2012) used lattice-gas cellular automaton models to determine that the rapid recurrence of gliomas post-resection cannot be explained by mutation theory alone, but tumors modeled with “go or grow” behavior can recapitulate the observed macroscopic growth patterns. Such models can even suggest treatment strategies, such as tumor oxygenation which encourages cells to revert to a proliferative and less radio- and chemo-resistant state. As Giatili and Stamatakos (2012) points out, discrete agent and cell based models are better suited to answer questions of the biological constitution of tumors over space and time. Continuum models give better insight to spatial extent and concentration profile of the population of tumor cells. Hatzikirou et al. (2012) showed that although the glioma cell population is heterogeneous and composed of significant portions of cells in both proliferative and migratory states, the microscopic simulation scales up to a reaction-diffusion model on the macroscopic scale practically identical to the Fisher equation.

### Turning mathematical predictions into mathematical neuro-oncology

**Figure 3:**
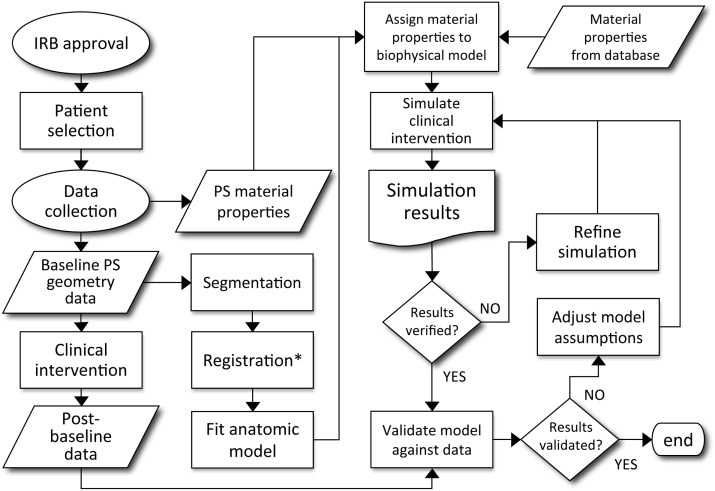
**Decision process for patient-specific model validation and translation to clinically applicable analysis**. Courtesy: Neal and Kerckhoffs (2010), by permission of Oxford University Press.

In a review of computational models of brain tumors Juffer et al. (2008) bemoans a “severe limitation of current models is that they are in fact *not* patient-specific at all.” However, mathematical models come in many forms and with different purposes. Some models aim to provide qualitative understanding or intuition regarding the phenomena of interest, while others are intended to provide predictions for specific scenarios. The effective use of the latter type of models depends on many factors: defining the quantity of interest, choosing the appropriate model, acquiring data for calibration, and then successfully subjecting the model to validation tests. By definition, patient-specific biological models require calibration for each patient. This inherently leaves room for philosophical debate regarding sufficient validation tests for patient-specific biological models, however, a good example for predictions involving clinical intervention is provided in Figure 3 (Neal and Kerckhoffs, 2010). One should note that this entire type of process would need to be redone for each possible application of the model.

As might be inferred from Figure 3, the cause of the “limitation” Juffer et al. (2008) raise is due to the difficulty of the entire process. The authors are only aware of work based on such an outline in the context of gliomas by the Swanson group (Swanson et al., 2002a, 2008b; Szeto et al., 2009b; Wang et al., 2009; Neal and Kerckhoffs, 2010; Rockne et al., 2010; Baldock et al., 2012a,b; Gu et al., 2012; Neal et al., 2012, 2013). But that is not to say that other efforts are not informative or useful. Indeed, many papers have been published (e.g., Zacharaki et al., 2009; Konukoglu et al., 2010), considering the formidable technical details involved in development and validation of a mathematical model that can be used to inform clinical decision making.

**Figure 4:**
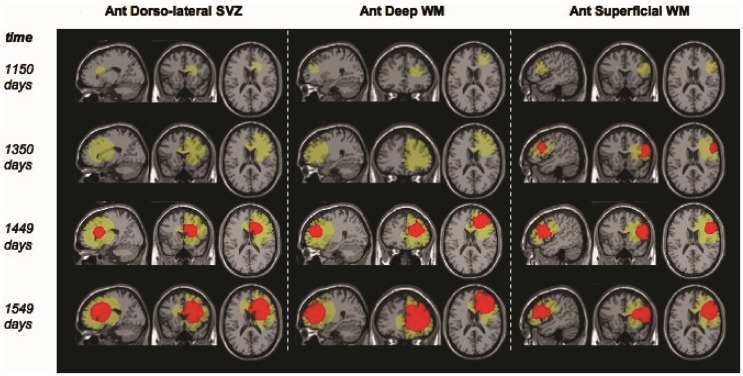
**“Example screenshots from glioma growth model simulations with varied points of origin**. Images at four time points each for three simulated lesions provided in the sagittal, coronal, and axial planes for lesion start points at the anterior dorsolateral subventricular zone, anterior deep white matter, and anterior superficial white matter. Green area reflects estimated T2-weighted image abnormality on magnetic resonance; red area reflects estimated T1-weighted image post-gadolinium abnormality.” Courtesy: Bohman et al. (2010).

Additionally, models may be used for qualitative understanding of events. An example of such work is that by Bohman et al. (2010) where they investigated ontogeny and spatio-temporal evolution of gliomas. By looking at a set of 63 patient tumors, they determined that tumors abutting the ventricle in the sub ventricular zone (SVZ) are larger than those that do not (Figure 4). The simulation results then pointed to an explanation in that two tumors with identical growth rates, as defined by the continuum mathematical model, could display markedly different growth patterns due to the anatomy of the brain and ontogeny of the tumor. Thus, it is not necessary for a mathematical model to be patient-specific to produce clinically significant results.

### Prognosis of individual patients using pre-treatment tumor growth kinetics

In a study of 32 newly diagnosed glioblastoma patients, Wang et al. (2009) used the PI model to find relationships between the Patient-specific model (PSM) parameters for glioma cell net dispersal (*D*), proliferation (ρ), and prognosis. As illustrated in Figure 1, patient-specific estimates *D* and ρ combine with the patient’s MRI to yield a map of the diffuse gradient of glioma cells that is expected to lie beyond thresholds visible to imaging (Figure 1). Wang et al. analyzed patient-specific tumor growth kinetics relative to the patients’ actual survival and found that the model parameters (specifically, ρ and ρ/*D*) were significant predictors of prognosis in both univariate and multivariate analyses even when controlling for standard clinical prognostic parameters such as RTOG recursive partitioning analysis (RPA) classification.

Velocity of radial expansion on MRI and net proliferation rates were compared to RPA classification and it was found that patients with low velocity and proliferation lived longer than the median prognosis associated with each RPA class, and patients with high velocity and proliferation had shorter survival. A therapeutic response index (TRI) was also calculated for each patient. This is defined as the ratio between the patient’s actual survival, and the time it takes for their untreated virtual control (UVC) tumor to reach fatal tumor burden (FTB) (Swanson, 2008; Wang et al., 2009). Patients with high rates of proliferation and velocity were found to have higher TRIs (Wang et al., 2009). This paper was perhaps the first in the literature for which a patient calibrated mathematical model for glioma growth generated prognostic parameters in a patient cohort.

### Quantifying tumor aggressiveness in individual patients

**Figure 5:**
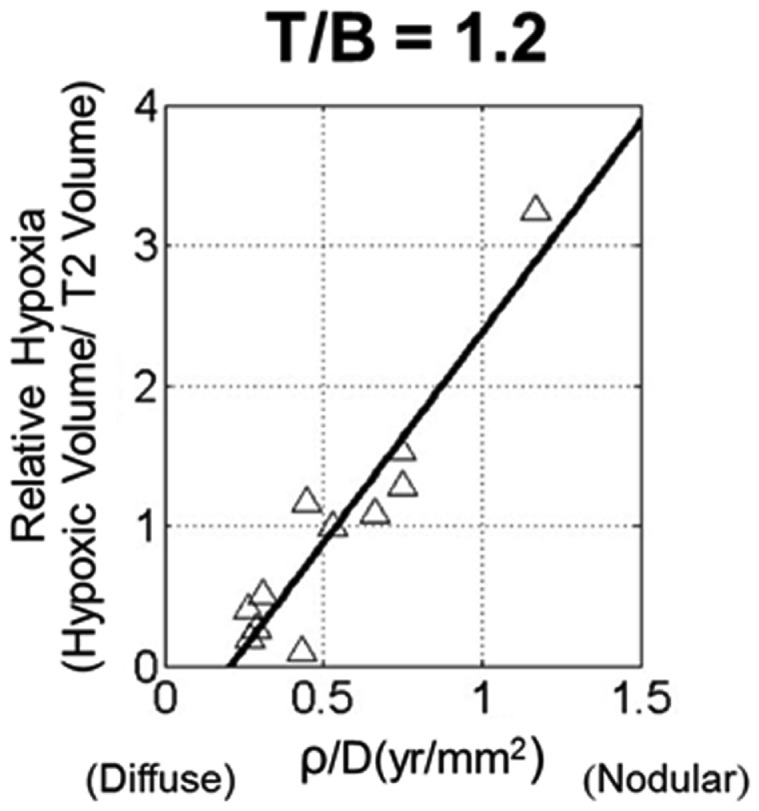
**Scatter plot of relative hypoxia (RH, the ratio of hypoxic volume to T2-weighted MRI volume) versus ρ/*D* for *n* = 11 glioblastoma patients**. RH was determined over a variety of tissue to blood (T/B) tracer levels, ranging from 1.1 to 1.6 in increments of 0.1. A strong linear relationship between the variables is shown for all thresholds; correlations were statistically significant for all T/B levels considered. From Szeto et al. (2009b), with permission from Nature Publishing Group, Cancer Research.

Despite the predictable pattern of linear radial growth in gliomas, within histologic grade there may be great variability in response to treatment and overall prognosis (Bonavia et al., 2011). The hypothesis that more aggressive tumors are more hypoxic was tested using PI model metrics of biological aggressiveness. Szeto et al. (2009b) found there was a strong relationship between hypoxia and the ratio of PI model parameters for proliferation and diffusion on 11 glioblastoma patients (Figure 5). Relative hypoxia (RH) was computed as the ratio of hypoxic volume obtained from pre-treatment ^18^F-Fluoromisonidazole (FMISO) PET images to the region of hyper intensity on T2-weighted MRI. They found that a tumor with high proliferation relative to diffusion would be a relatively well demarcated lesion, while a low ratio would indicate a very diffuse tumor with more migratory capacity compared to the proliferation rate. A metric of tumor shape irregularity was also calculated and found to be negatively correlated with ρ/*D*. This suggests that more irregularly shaped tumors are formed by cells with relatively high proliferation rates in highly hypoxic environments. These metrics yield patient-specific understanding and quantification of disease burden and relative biological aggressiveness and a tool in the MNO toolbox.

### Going beyond the routine: Advanced imaging in mathematical modeling

Extending the PI spatio-temporal model of glioma proliferation and invasion, Swanson’s group (Swanson et al., 2011; Gu et al., 2012) incorporated neoangiogenesis-a defining hallmark of high grade glioma into the PI model. Briefly, this Proliferation-Invasion-Hypoxia-Necrosis-Angiogenesis (PIHNA) model includes invading normoxic glioma cells which become hypoxic when local resources are exhausted. This results in the local production of significant amounts of angiogenic factors that, in turn, stimulate an angiogenic response. If the angiogenic response is sufficiently robust, these hypoxic cells may revert to normoxia; however, if the angiogenic response is insufficient then necrosis may result. Patient-specific simulations of this type allow for the generation of spatio-temporal maps of normoxic cells, hypoxic cells, necrotic tissue, vascular volume fraction, and angiogenic factors.

**Figure 6:**
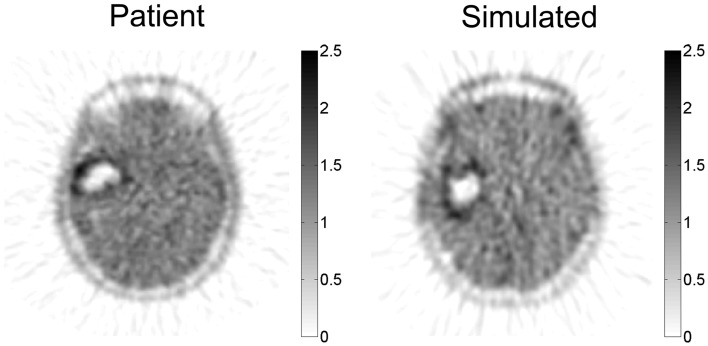
**Simulated FMISO-PET and actual FMISO-PET**. Hypoxia is predicted by the PIHNA model and an imaging reconstruction algorithm produces the simulated FMISO-PET. Pixel intensity distribution is not statistically different between the two images, providing model-based predictions of tumor hypoxia which is otherwise obscured by PET image acquisition and reconstruction. Courtesy: Gu et al. (2012), by permission of Oxford University Press.

The PIHNA model predicts a patient-specific spatial map of hypoxia, which can be compared with PET imaging with the hypoxia tracer ^18^F-FMISO (Gu et al., 2012). Since there is significant image noise introduced from PET image acquisition and reconstruction, a combination of a pharmacokinetic model for the FMISO tracer kinetics and an image reconstruction algorithm for PET were applied to the patient-specific simulated hypoxic cell distribution to generate a patient-specific *in silico* PET image with striking similarity to the patient’s actual image (Figure 6).

### The untreated virtual control

**Figure 7:**
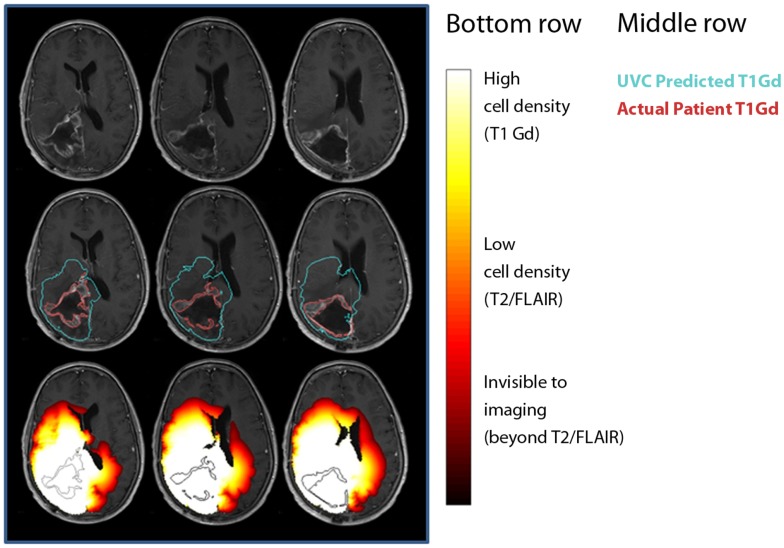
**Comparisons between untreated virtual controls and post-treatment MRI scans**. First row: post-treatment MRI. Second row: contours showing measured tumor on T1-Gd-enhanced scan (red) and UVC prediction of T1-Gd area (aqua). Third row: UVC tumor cell densities overlaid (white, high cell density; red, low cell density) on scan with T1-Gd measured tumor outline (black).

To date, the most effective demonstration of the clinical utility of mathematical modeling has been in the context of UVC (Figure 7) (Swanson, 2008; Wang et al., 2009). The concept of an UVC is that a model that accurately describes the inherent, untreated disease behavior as a baseline for future comparisons for a specific patient. Deviations from the predicted “control” behavior can be assessed and used as a metric of response to therapy. Because gliomas have a simple, predictable pattern of untreated growth, the UVC approach is particularly simple to apply in this case.

## Treatment Response and Optimization

Few treatment options exist for newly diagnosed glioma beyond surgery and chemoradiation following the landmark study which established standard of care for the disease (Stupp et al., 2007). Novel therapies are often reserved for the recurrent setting and have shown little benefit in prolonging survival. Due to the relative rarity of the disease, powering clinical studies can be challenging. Mathematical models quantifying response, sensitivity, and relative benefit of treatment (UVC) provide a novel and alternative means of stratifying patients for clinical studies.

### Days gained score as a treatment response metric

The Days Gained score provides a measure of patient-specific treatment derived benefit in terms of treatment induced deflection in tumor growth from the UVC (Neal et al., 2013). This novel quantification stands in stark contrast to current metrics of response (e.g., Macdonald criteria, RANO, and RECIST) that do not account for the relative growth kinetics of individual tumors. One clear difference is that static imaging-based metrics allow a poor response for a slow growing tumor to be equated with a significant response from a fast growing tumor. Neal has shown that the Days Gained metric indeed performs better than these existing metrics in determining patients that will have a survival benefit from treatment. While these classic response criteria are actively being reconsidered in the context of gliomas (Wen et al., 2010), the UVC PSM represents an opportunity to incorporate the implicit heterogeneity of glioma growth kinetics across patients into measures of treatment response.

### Surgical resection

**Figure 8:**
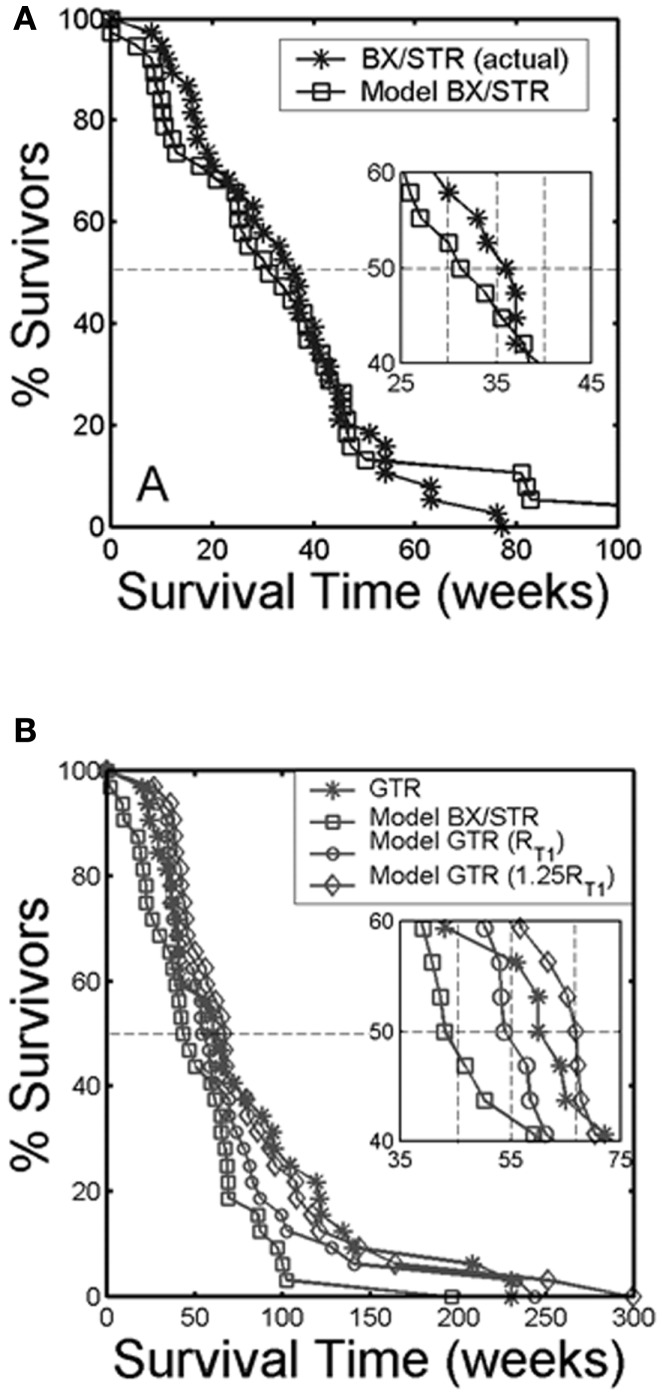
“**(A)** Survival curves for actual glioblastoma patients (asterisks) and virtual patients (squares) subjected to biopsy or subtotal resection (BX/STR, *N* = 38). Inset shows a close-up of the survival curves near the median survival times of 32.4 and 36.5 weeks. **(B)** Survival curves on a longer time scale following gross total resection (GTR, *N* = 32) in actual patients (asterisks) defined by the absence of residual tumor on post-operative enhanced CT. The virtual patients (matched to actual pre-operative T1-Gd volume and *D*/ρ ratio derived from the T1-Gd and T2 volumes) were subjected to no resection (BX/STR, squares), to resection of 100% of the T1-Gd volumes or radii, rT1 (circles), and to resection of 125% of the T1-Gd volumes or radii, 1.25 rT1 (diamonds). Inset shows a close-up of the survival curves near the median survival times of 44.9, 55, 62, and 66.9 weeks.” Reprinted from Swanson et al. (2008b) with permission from Nature Publishing Group, British Journal of Cancer.

Surgical resection is the first line response to clinical presentation and radiographic diagnosis of a malignant brain tumor. Although the survival benefit of subtotal (STR) versus gross total (GTR) removal of imageable tumor remains controversial, Swanson et al. (2008b) used mathematical modeling to simulate surgical resection using the PI model for 70 glioblastoma patients using contrast-enhanced T1-weighted MRI volume and radial velocity of tumor growth. The model was able to predict the survival curve for biopsy and subtotal resection groups (Figure 8A). Simulations were performed to represent 100 and 125% resections, the observed gross total resection survival curve was found to lie between these two virtual curves (Figure 8B). These results suggest that although GTR provides a survival benefit over patients receiving biopsies or subtotal resections, this is partially due to the preferential selection of patients with smaller tumors for gross total resection. This analysis, made possible with mathematical modeling, provides valuable insight into a controversial clinical debate.

### Quantifying and predicting response to radiation therapy

**Figure 9:**
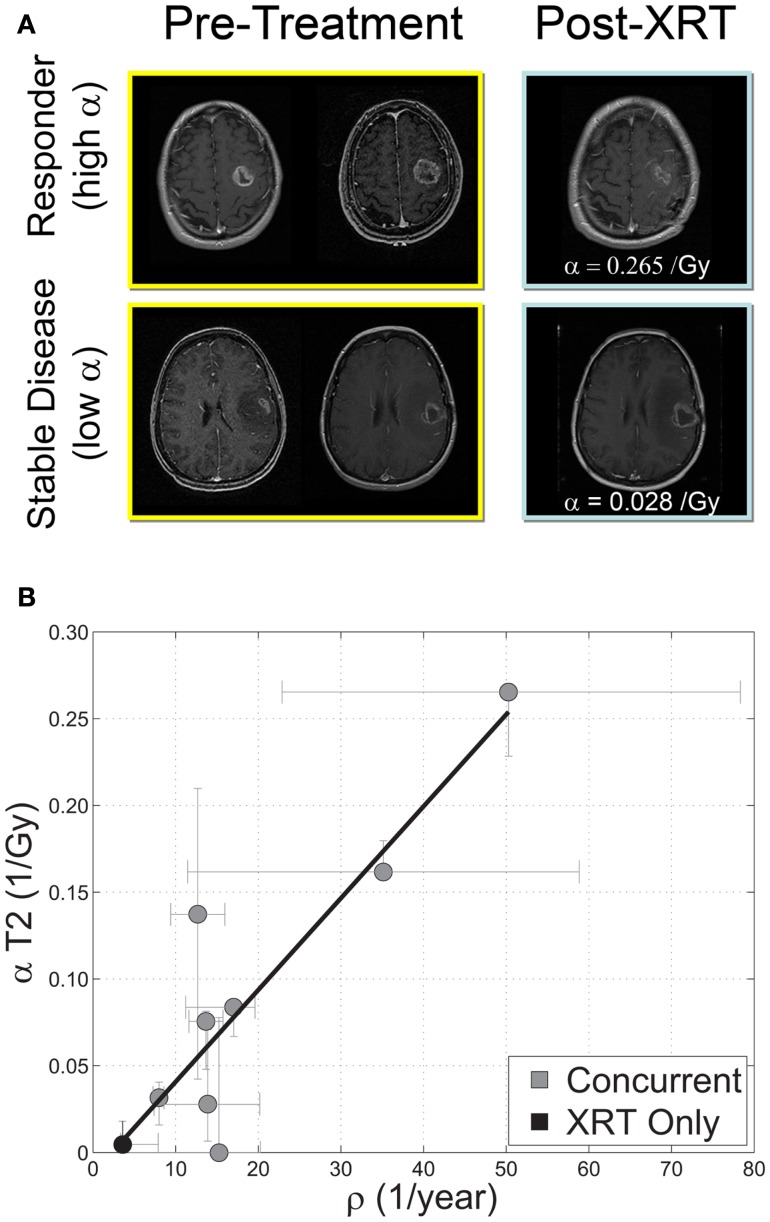
**(A)** “Response to therapy is conventionally assessed by determining changes in gross tumor volume (GTV) on MRI prior to and after the administration of therapy. Post-contrast T1-weighted MRI images are shown for two glioblastoma patients that would typically be separated into generic groups: responder and stable disease. The radiation response parameter α gives an additional quantification of radiation response for each patient.” **(B)** “Relationship between radiation response and tumor proliferation rate parameters α (Gy^−1^) and ρ (1/year), respectively, with α calculated relative to changes in T2 GTV post therapy *r* = 0.89, ρ ≪ 0.05, *N* = 9. Error bars on ρ are calculated by propagation of error in pre-treatment GTV as assessed by inter-observer variability of ±1 mm in equivalent spherical radius. Error bars in α are computed by taking the maximum and minimum values of α in a leave one out cross validation (LOOCV) technique.” Courtesy: Rockne et al. (2010), with permission from IOP Publishing Ltd.

Beyond RECIST and Macdonald response criteria (Padhani and Ollivier, 2001; Galanis et al., 2006; Therasse et al., 2006), quantifying the *in vivo* biological effectiveness of radiotherapy in individual patients has remained elusive (Enderling et al., 2010). Rockne et al. (2009, 2010) incorporated the classic linear-quadratic model for radiation effectiveness (Bauman et al., 1999; Sachs et al., 2001) into the PI model to quantify the effectiveness of radiotherapy in individual glioma patients. The extended model (PIRT) uses radiation dose plans from the clinical treatment system and fractionation – e.g., 1.8 Gy fractions delivered to the T2 abnormality with a 2.5-cm margin. Nine glioblastoma patients with two MRIs before the initiation of radiotherapy and at least one MR after the completion of radiation therapy were included in the study. The authors found a strong correlation between the net proliferation rate (ρ) of the glioma cells before the initiation of treatment and the radiation effectiveness (Figure 9). The predictive precision of this relationship was tested with a leave one out cross validation (LOOCV) analysis which revealed an average 2.4 mm difference between simulated and actual tumor volume post RT which given an average GBM radius of 2 cm represents a relative error of at most 15%. The error is substantially more resolved than the 25% categories presented in RECIST or Macdonald criteria (Padhani and Ollivier, 2001; Therasse et al., 2006). This approach has provided the first *in vivo* quantification of radiosensitivity in individual glioma patients as well as a predictive relationship between pre-treatment growth kinetics and response to therapy.

*In silico* models of tumor growth and response to radiotherapy allow for the investigation of factors affecting radiosensitivity and alternative treatment strategies that may be impractical in the clinic. Stamatakos et al. (2006) model studies the interdependent effects of oxygenation on radiosensitivity, angiogenesis, and clonogenic cell density on tumor growth. Lower oxygen enhancement ratio and lower clonogenic cell density were among the factors found to increase radiosensitivity, agreeing with clinical experience, although not directly compared with clinical data. Both Powathil et al. (2007) and Rockne et al. (2009) used a continuum reaction-diffusion model along with the classic linear-quadratic model for radiotherapy effect to investigate alternative fractionation strategies on a virtual tumor with fixed tumor growth kinetics and radio sensitivity. To date, neither model has incorporated the toxic effects of radiation on normal tissue. Holdsworth et al. (2012) builds upon these foundations by refining intensity-modulated radiation therapy (IMRT) plans based on the criteria of maximizing cytotoxicity while minimizing normal tissue dose.

Swanson et al. (2008a) used the concept of a UVC to understand relative treatment response effects on individual survival time assuming a FTB (Concannon et al., 1960). Results of this investigation demonstrate that patient-specific rates of invasion and proliferation as estimated by a reaction-diffusion model can be calculated for individual patients and related to radio-resistance or radiosensitivity in individual patients and that the mathematical model can be used to determine radio efficacy by relating survival times predicted by the UVC to that observed in the patients, assuming a FTB. In this population, Swanson et al. were able to identify those patients that benefited significantly from radiotherapy by comparing model-predicted untreated survival time with actual (treated) survival time.

### Optimizing radiation therapy

*In silico* models of tumor growth and response to radiotherapy allow for the investigation of alternative treatment strategies that may be impractical in the clinic. Holdsworth et al. (2012) leveraged the patient-specific description of tumor growth and response in the PIRT model (Rockne et al., 2010) to generate biologically guided treatment plans. Using an adaptive, multiobjective evolutionary algorithm (MOEA), IMRT plans were optimized with respect to a variety of clinical objectives including maximizing normal tissue sparing and minimizing the tumor burden at various time points. By using the PIRT model-predicted tumor burden 12 weeks post-irradiation as an optimization objective for each week of simulated treatment, the MOEA computed radiotherapy plans that improved treatment gain by an average of 122.5 days and reduced equivalent uniform dose (EUD) to normal tissue an average of 15.5 Gy for two example patients (Holdsworth et al., 2012).

### Predicting pseudoprogression

Pseudoprogression is a puzzling clinical phenomenon defined by increased contrast enhancement on MRI within 100 days of radiation therapy that spontaneously improves with no subsequent change in treatment (Brandsma et al., 2008). It has been estimated that 20–47% of tumors exhibiting increased contrast enhancement on MRI within 12 weeks following chemoradiotherapy are not indicative of true progressive disease, but are a result of pseudoprogression (Brandsma et al., 2008; Clarke and Chang, 2009). This poses a significant clinical challenge as the current standard of care for recurrent glioma disease calls for immediate changes to chemotherapeutic regimens upon clinical assessment of tumor progression as indicated by increased contrast enhancement on MRI. In addition, recurrent glioma disease is often treated with a second surgical resection of the T1-weighted gadolinium enhanced region (Stupp et al., 2010). Although there is currently no understanding of the underlying biological mechanisms to understand and predict which patients will exhibit pseudoprogression, the Days Gained metric has been shown to discriminate pseudoprogression from true progression in individual patients (Neal et al., 2013). Patients with pseudoprogression were found to have significantly higher Days Gained scores, connecting model-based metrics of response to clinical outcomes in individual patients.

## Summary

Gliomas present a unique clinical challenge. In addition to intra- and inter-tumoral heterogeneity, these lesions are defined by their diffuse invasion of otherwise normal-appearing brain tissue peripheral to the imageable abnormality. This diffuse growth limits the clinical utility of neuroimaging in interpreting treatment response. Current metrics of therapeutic response rely on observable changes to clinical imaging (Wen et al., 2010), ignoring the underlying growth dynamics of the tumor. Further, the current standard of care leaves few treatment options and may over-treat patients with slow growing tumors. Patient-specific mathematical modeling provides a novel means of developing UVCs for each patient’s tumor and provides predictive insight into prognosis, treatment response, and optimal treatment design.

The future of patient-specific modeling and application depends on asking questions that mathematical models can realistically answer with data that can be obtained from patients either non-invasively or infrequently. PSM must be validated and incorporated into clinical trials to become broadly and directly applicable to patient care. Advantages of a patient-specific modeling approach include:

○ Identification of individualized tumor proliferation and invasion rates or other kinetic information about an individual patient’s tumor (Swanson et al., 2002b; Mandonnet et al., 2003; Pallud et al., 2006; Harpold et al., 2007; Swanson, 2008; Szeto et al., 2009a,b; Wang et al., 2009; Boone et al., 2010; Rockne et al., 2010; Gu et al., 2012)
○ Development of methods for quantifying and predicting response to therapy – alone and also with respect to UVCs provided by model predictions (Swanson et al., 2008a; Wang et al., 2009; Rockne et al., 2010)
○ More informed treatment planning and response assessment tools that compare each patient’s tumor growth against its own virtual control (Swanson et al., 2002a, 2008a,b; Harpold et al., 2007; Szeto et al., 2009b; Wang et al., 2009; Rockne et al., 2010; Gu et al., 2012)

These advantages directly address a number of key unmet challenges in clinical neuro-oncology. In the coming years we anticipate a continued expansion of peer-reviewed journals dedicated to mathematical oncology, coordinated with increased funding for research in the area. Recently, Cancer Research has added a special section devoted exclusively to mathematical oncology, and the NIH has initiated special funding programs targeted at mathematical models through the Integrative Cancer Biology Program and Physical Sciences Oncology Center, among others. By producing individualized virtual tumors that predict disease progression in the absence of treatment, patient-specific modeling can contribute to the ongoing dialog regarding the design of appropriate response criteria (Wen et al., 2010), provide a means to perform virtual clinical trials to assess the likely benefit of novel neurotherapeutics, and move neuro-oncology toward individualized treatment plans optimized for maximum benefit.

## Conflict of Interest Statement

The authors declare that the research was conducted in the absence of any commercial or financial relationships that could be construed as a potential conflict of interest.

## Abbreviations

D, net rate of diffusion; FTB, fatal tumor burden; MM, mathematical modeling; MNO, mathematical neuro-oncology; MRI, magnetic resonance imaging; PSM, patient-specific modeling; ρ, net rate of proliferation; UVC, untreated virtual control.
